# Understanding of metals dysregulation in patients with systolic and diastolic dysfunction in ischemic heart disease

**DOI:** 10.1038/s41598-020-70733-4

**Published:** 2020-08-18

**Authors:** Noman Khan, Satwat Hashmi, Amna Jabbar Siddiqui, Sabiha Farooq, Shahid Ahmed Sami, Nageeb Basir, Syeda Saira Bokhari, Hasanat Sharif, Sanaullah Junejo, Hesham R. El-Seedi, Syed Ghulam Musharraf

**Affiliations:** 1 H.E.J. Research Institute of Chemistry, International Center for Chemical and Biological Sciences, University of Karachi, Karachi, 75270 Pakistan; 2 Department of Biological and Biomedical Sciences, Agha Khan University, Karachi, 74800 Pakistan; 3 Dr. Panjwani Center for Molecular Medicine and Drug Research, International Center for Chemical and Biological Sciences, University of Karachi, Karachi, 75270 Pakistan; 4 Department of Surgery, Aga Khan University, Karachi, 74800 Pakistan; 5 Department of Medicine, Aga Khan University, Karachi, 74800 Pakistan; 6 South City Hospital Karachi, Karachi, 75600 Pakistan; 7 Pharmacognosoy Group, Department of Medicinal Chemistry, Biomedical Centre, Uppsala University, Box 574, 75 123 Uppsala, Sweden; 8 International Research Center for Food Nutrition and Safety, Jiangsu University, Zhenjiang, 212013 China

**Keywords:** Cardiology, Cardiovascular biology, Heart failure, Analytical chemistry, Biochemistry

## Abstract

Ischemic heart disease (IHD) is the leading cause of death and chronic disability in the world. IHD affects both the systolic and diastolic function of the heart which progressively leads to heart failure; a structural and functional impairment of filling or ejection of blood from the heart. In this study, the progression of systolic and diastolic dysfunction characterized according to their echocardiographic parameters including left ventricular ejection fraction (EF), grades of diastolic dysfunction and ratio between early mitral inflow velocity and mitral annular early diastolic velocity (E/eʹ), were correlated with differential regulation of various metals in patients sera samples (n = 62) using inductive coupled plasma-mass spectrometry (ICP-MS). Chromium, nickel and selenium were found significant (p < 0.05) in patients having EF < 45% compared with EF > 45%. In patients with systolic dysfunction (EF < 45%), the level of selenium was decreased while the level of chromium and nickel was increased compared to patients with EF > 45%. Selenium level was also decreased significantly (p < 0.05) in grade 1A and 2 patients that are considered as higher grades of diastole dysfunction in comparison to grade 0–1. Overall, selenium deficiency was identified in both systolic and diastolic dysfunctions of IHD patients corresponding to the progression of disease that could be related to many metabolic and translational pathways specifically which involve selenoproteins.

## Introduction

Cardiovascular diseases (CVDs) are the leading cause of death worldwide^1^. An estimated 17.9 million people died from CVDs in 2015, representing 31% of all global deaths. Ischemic heart disease (IHD) account for the majority of the cases of health lost to CVDs. There are large number of prevalent cases of IHD making it the leading cause of chronic disability^1,2^. The most common cause of IHD is atherosclerosis which is a chronic inflammatory process represented by highly specific cellular and molecular responses^3^. IHD affects both the systolic and diastolic function of the heart which progressively leads to heart failure; a structural and functional impairment of filling or ejection of blood from the heart^4^. Despite advances in the current treatment plans, heart failure remains one of the major health care issues of the world not only due to its increasing prevalence but also health care costs^5^.

Development of high-throughput technologies for the generation of ‘omics data’ have been at the forefront to understand the development of disease^6^. Metallomics, an emerging branch of omics, deals with a comprehensive analysis of all the metals and metalloid species within a cell or tissue^7,8^. It is an important field enabling to understand the relationship between metals and their biological and physiological functions, identification and quantification of these trace elements in association with the disease diagnosis and progression.

Few studies have reported the quantification of specific metals in whole blood and serum samples of ischemic heart disease^9–12^. However, these studies did not involve any clinical correlation with metal concentrations. In our study, we employed a correlation of metallomic analysis with patients categorized on the basis of their systole function i.e. EF < 45% and EF > 45%. Development of diastolic dysfunction was also studied using other echocardiographic parameters that are grades of diastolic dysfunction (0–1, 1A and 2) and ratio between early mitral inflow velocity and mitral annular early diastolic velocity, E/eʹ (< 8, 9–15 and > 15) of IHD patients in order to gain insight into metallomic status of patients in understanding of disease progression.

## Material and methods

### Patient’s selection and classification

This study was recruited IHD patient, which were admitted for coronary artery bypass grafting (CABG) in South City and Aga Khan University Hospital of Karachi. The samples were collected from both hospitals after obtaining written informed consent from the patients. The ethical approval boards of South City, Aga Khan University Hospital and Institutional Review Board (IRB) of ICCBS approved the current study. All methods were performed in accordance with the relevant guideline and regulations approved by the committee. The assessment of systolic and diastolic function with left ventricular (LV) morphology was conducted by 2D Doppler echocardiography according to the guideline of ASE 2009.

Patients were classified into different categories according to the ejection fraction (EF < 45% and EF > 45%) and diastolic dysfunction by E/e values (> 15, 9–15 and < 8) and grades (0–1, 1A and 2) as depicted in Table 1. It was observed that the corrected *p*-values are significant for few patients` basic characteristics. Hence an unsupervised learning algorithm was applied on the concentrations of metals with reference to the age, weight, BMI and blood pressure (both systole and diastole). The results in the form of PCA score plot are shown in Supplementary Figure S1. No significant separation trend/grouping was observed in all plots. Dysfunction of diastole^13,14^ were categorized with different grades include impaired relaxation of LV with or without increased in filling pressure indicated by grade I or IA, moderate increased in LV filling pressure is linked with the pseudo-normalization of LV i.e. grade II and increased in filling pressure markedly is the restrictive LV filling denoted as grade III. Patients with malignancy, constrictive pericarditis and infiltrative, established pulmonary disease, renal insufficiency, moderate to severe valvular disease, hypertrophic cardiomyopathies and metabolic bone diseases were omitted from the study It is possible that the screened biochemical parameters are influenced by the cardiac dysfunction either systole or diastole. Therefore, we tested this hypothesis and results are incorporated in Supplementary Table 1. It was clearly observed from the table results that the biochemical parameters of the recruited patients are not statistically significant different with reference to the ejection fraction values and grades.

**Table 1:** Experimental subject description of healthy and ischemic heart disease (diastolic and systolic) of serum samples.

Patient characteristics	Disease	Healthy	p-value
Number of samples	62	55	–
Gender (male/female)	54/8	44/11	–
Age male (years; µ ± σ)	58.90 ± 8.34	31.3 ± 6.41	2 × 10^–33^
Age female (years; µ ± σ)	58.12 ± 9.51	30.18 ± 9.03	3 × 10^–5^
Height (cm)	163.80 ± 8.91	167.32 ± 7.95	0.0649
Body weight (Kg)	72.41 ± 11.94	65.49 ± 6.99	8 × 10^–5^
BMI (Kg/m^2^)	26.66 ± 4.83	23.59 ± 3.64	0.0007
SBP (mm Hg)	129.46 ± 18.60	112.91 ± 10.48	1 × 10^–7^
DBP (mm Hg)	75.67 ± 9.69	72.27 ± 8.65	0.0394
**Medical history**	**Frequency**		
Diabetes/non-diabetes	37/25	0/55	
Hypertension/non-hypertension	41/21	0/55	
Chest pain/non-chest pain	48/14	0/55	
Current smoker/non-smokers	11/51	0/55	
Hypercholesterolemia/non-hypercholesterolemia	6/56	0/55	
**Echocardiographic parameters**			
Ejection fraction (< 45%/ > 45%)	12/50	–	
Grades 0–1/1A and 2)	45/4/and 5	–	
E/e′ (< 8/9–15 and > 15)	12/33 and 7	–	
**Biochemical parameters**			
Creatinine (mg/dl)	0.99 ± 0.26	–	
BUN (mg/dl)	17.74 ± 7.85	–	
Random blood glucose (mg/dl)	183.67 ± 64.81	–	
Fasting blood glucose (mg/dl)	164.96 ± 57.98	–	

*µ* mean, *σ* standard deviation, *BMI* body mass index, *SBP* systolic blood pressure, *DBP* diastolic blood pressure, *BUN* blood urea nitrogen.

Healthy control subjects (n = 55) were selected based on the following criteria; subjects were recruited randomly from the community if they had no symptoms suggestive of IHD, no past history of IHD or any proven past myocardial infarction or coronary intervention and without any symptoms of functional class I.

### Sample collection and processing

BD vacutainer tube based on gel (Cat # 367381), was used to transfer 4 ml of blood, which was collected from the subject under investigation. After standing the BD vacutainer tube for 10–15 min, serum samples were separated by using centrifugation at 2000 rpm for 10 min. After centrifugation, aliquots of serum were transferred into locking Eppendorf tube. Serum samples were kept in freezer at − 80 °C until sample analysis.

### Reagents and standards

During the experimental work, filtered and extremely pure deionized water was obtained from the water filtration and purification system (Thermo scientific, MA USA). Analytical grade reagent (AR, ACS), 70% concentrated HNO_3_ (RCI Labscan Ltd, Bangkok, Thailand) was used for analysis after purification by NanoPure Acid purification system (Nanonex, USA). Trace metal grade ≥ 30% concentrated H_2_O_2_ was acquired from Merck KGaA company (Darmstadt, Germany). The tuning solution with 1 µg/L concentration of Mg, Li, Tl, Y, Co and Ce in 2% HNO_3_, multi-element calibration solution 2A (Part Number: 8500-6940) with confirmed concentration of 10 µg/mL of each element and, internal standard of 100 µg/mL (Tb, Rh, Ge, Lu, Ir, Bi, and Sc) were bought from Agilent Technologies (Santa Clara, CA, USA). The optimization of ICP-MS parameter was carried out by using tuning solution, before the start of analysis. All cleaned glassware and polypropylene bottles were immersed in 10% (v/v) HNO_3_ reagent for overnight. After that each apparatus was washed with ultra-pure water three times and kept in laminar-flow hood (Airstream ESCO, Singapore) to dry. The air contamination was prevented by performing experiment in clean hood and working table.

### Preparation of standard solutions

Matrix solution prepared as 5% nitric acid was used to prepare calibration standards for 16 elements. The 100 µg/L solution of internal standard was made in matrix solution, from the prepared stock solution. Sixteen points calibration curves were prepared in the range of 0.0076–1,000 µg/L of each metal using 5% HNO_3_ as matrix. A blank was also prepared with same matrix and no metal added. The measurement of sensitivity was done by checking the slope of regression equation. The standard solution was used to validate the current study by calculating correlation coefficients, LOD and LOQ.

### Preparation of the standard reference material

The developed method was validated by measuring precision and accuracy of the result obtained from trace metal Seronorm serum L-1 (Sero, Billingstad, Norway). The complete procedure for preparation of certified reference material (CRM) was followed as given in the protocol of manufacturer. For preparation, 3 mL of sterile deionized water was added to dissolve the content of vial by rolling for 30 min so that all the content is mixed completely. The content was transferred to screw cap plastic tube and diluted with sterile deionized water. Each trace and ultra-trace element were analysed in triplicate in diluted CRM samples by ICP-MS.

### Sample preparation for ICP-MS

The digestion of serum samples were carried out in pressure sealed microwave system with 64MG5-T64 rotor (Anton Paar GmbH, Austria). It was equipped with high performance pressure released system. The program with Multiwave ECO software (version 1.51), was setup in the microwave system. The single time useable screw cap (13–425, Wheaton 15 × 45 mm) and polytetrafloroethylene (PTFE) lip seal tube were used as standard apparatus. For digestion, serum sample equivalent to 50 µL aliquot was added in the MG5 vials (Anton Paar, Hungary), where 50 µL of ≥ 30% H_2_O_2_ and 150 µL of 70% HNO_3_ were mixed, kept in laminar fuming hood for 15 min so that fumes were evolve from the vials. Then, vials were sealed with the PTFE lip and screw cap. After that sealed vials containing serum samples were placed and digested in two steps by adjusting same parameters in Anton Paar microwave system with same ramp (10.0 min), hold (30), fan (1), power (850), stir rate (medium) but temperature set 90 °C in step 1 and 150 °C in step 2. All MG5 vials were kept in laminar hood and wait until all samples were cooled at room temperature, as digestion process completed. The vials were sealed with septum so that pressure of gas released by penetrating steal pin in septum then, the resultant samples were took into 15 mL of autosampler polypropylene tubes and deionized water were used to dilute samples upto 3 mL. All the samples were analysed in triplicate for every element. Matrix correction was done by using internal standard solution, to check the accuracy of ICP-MS results.

### Inductive coupled plasma–mass spectrometry (ICP-MS) analysis

The quantification of chosen element was carried by Agilent 7,700 × ICP-MS system (Santa Clara, CA USA). The decontamination was removed from ICP-MS after each sample analysis by using washing solution contains 0.1% HCl, 2% HNO_3_ and ultra-pure water. The workstation used for the operation of ICP-MS data is Mass Hunter software. ICP-MS parameter are given in the Supplementary Table 2.

### Statistical analysis

The chemometric analysis was done in multiple steps by Mass Profiler Professional (MPP), which were bought Agilent technologies (Santa Clara, CA, USA) with complete licenced. The absolute abundance was adjusted with 10,000 counts for filtration process. The normalization of data was carried out by external scalar to allocate the values for each sample particularly scale up and down. The selection of baseline i.e. Z transform, is important to treat all components equally, whatever their intensity.

Statistical analyses were performed in-between healthy and ischemic heart disease patient samples; characterized by systolic dysfunction by ejection fraction < 45% and > 45%, and diastolic dysfunction by grades and E/eʹ. Unpaired t test was used for statistical analysis of two groups while ANOVA was employed for more than two groups’ analyses. p-value was calculated by Asymptotic computation method and Benjamini–Hochberg FDR was used for multiple test corrections. All variables with p values < 0.05 and fold change > 1.5 will be considered as significant variables throughout the manuscript.

Multivariate data analyses including unsupervised principal component analysis (PCA) (shows an overview and outlier behaviour) and supervised partial least squares discriminant analysis (PLS-DA) and orthogonal partial least squares discriminant analysis (OPLSDA) were performed on processed data by SIMCA MKS Umetrics AB (version 14.1) software.

### Method validation and data quality assessment

Linear calibration curve was obtained in the concentration range of 0.0076–1,000 µg/L for every element. The least-square regression method was used to plot the data of counts per second (cps) against the measured concentrations. In the validity of the quantification method was evaluated by performing various parameters such as correlation coefficients (R^2^), limit of quantification (LOQ) and limit of detection (LOD). The excellent linear relationship was obtained from the calibration curve (n = 3) with correlation coefficients (R^2^) between 0.993 and 1.000. LOQ and LOD was calculated using equation LOD = 3.3σ/S and LOQ = 10σ/S, where (σ) standard deviation of residual a regression line and (S) slope for each element. LOD and LOQ were found for all selected element in the range of 0.002–9.551 µg L^−1^ and 0.006–28.941 µg L^−1^ respectively. Supplementary Table 3 compiled the regression equation, R^2^, LOD and LOQ of each element.

To check the precision and accuracy for the developed method, Seronorm trace elements serum L-1 was used as reference standard material (CRM) for trace metal analysis in bio samples. It was found that all observed values agree with the certified values with nonsignificant variance between them. The % recovery of selected element was varied between 81.879 and 113.779, which are given in Supplementary Table 4.

For the assessment and validation of any analytical technique, it is necessary to perform spike recovery procedure. The analyte detection was monitored by checking the variation between the diluent used to prepare the sample and standard solution. The spike recovery test was also performed in real serum samples of two concentration levels for each element, to check the reliability and precisions (RSD, %) of our method. Mostly, the precision was found below 10%. The following relationship used to measure the precision of the method, Precision (RSD, %) = [standard deviation (SD)/CM] 100, where CM for measured concentration and SD means standard deviation. For serum samples, the coefficient of variation (% RSD) was obtained from 0.052 to 7.580 as shown in Supplementary Table 5. Thus, our method, is sensitive, accurate with good precision and can be employed for the routine metals analysis in biological samples.

### Compliance with ethical standards

All procedures performed in studies involving human participants were in accordance with the ethical standards of the institutional and/or national research committee and with the 1964 Helsinki declaration and its later amendments or comparable ethical standards.

## Results

### Metallomic fingerprinting of serum for IHD patients and healthy subjects

ICP-MS analysis of serum samples for IHD patients and healthy subjects was performed for a total of 16 elements. Using absolute concentrations of elements, nine out of sixteen were found to be significantly different (p < 0.05) up regulated or down regulated between serum of healthy subjects and IHD patients (Table 2). Four elements including selenium, lithium, aluminum, copper were down regulated and five element including zinc, silver, arsenic, cadmium and manganese up regulated in serum of IHD patients in comparison to healthy controls.

**Table 2:** List of elements in serum of IHD patients that are significantly different from serum of normal healthy subjects.

Elements	p (Corr)	Log FC (SR IHD vs SR Healthy)	Regulation (SR IHD vs SR Healthy)
7 Li	2.91 × 10^–13^	− 1.18567	Down
78 Se	7.11 × 10^–15^	− 0.76438	Down
27 Al	8.00 × 10^–04^	− 0.49217	Down
63 Cu	0.001468	− 0.41605	Down
55 Mn	0.015824	0.227213	Up
66 Zn	1.05 × 10^–07^	0.785	Up
75 As	2.27 × 10^–05^	0.447256	Up
107 Ag	8.76 × 10^–05^	0.392908	Up
111 Cd	0.011826	0.125726	Up

**Figure 1:**
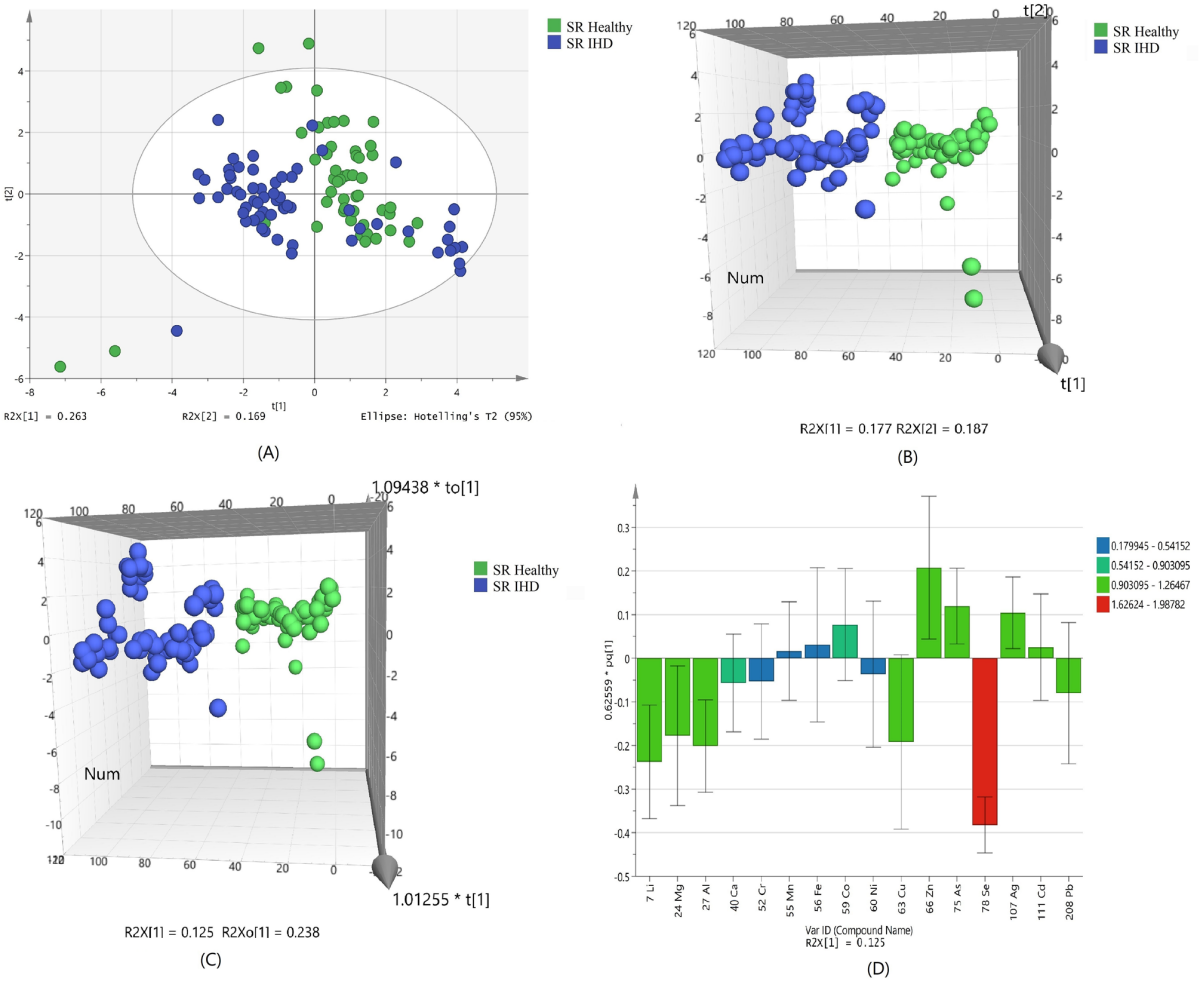
Scores scatter plots (**A**) PCA, (**B**) 3D PLS-DA, (**C**) 3D OPLS-DA and (**D**) OPLS-DA loadings plot colored as a function of VIP of serum (SR) from healthy (green) and ischemic heart disease (IHD) (blue).

Based on the normalized concentration of elements, a clear trend of separation between IHD patient and healthy subjects was observed in PCA score plot (Fig. 1A). The first component at x-axis gave the value of R_2_X 0.263 while the second component is 0.169. Confidence limit of 95% from Hotelling’s T2-test resulted in appearance of few outliers, which were further removed before discriminant analyses. Variations of few samples were resulted in their PCA scores equivalent to the values that are near to the opposite group, but did not found any clinical correlation among these samples.

Using the normalized concentrations of nine significant metals for class discrimination analysis, it was found that healthy subjected were clearly separated from IHD patients as shown in the 3D score plot of PLSDA (Fig. 1B). The number of patients, which were correctly predicted as true positives, was used to calculate the sensitivity; while the number of healthy, which were correctly predicted as true negative, was used to calculate the specificity for the constructed model. The specificity and sensitivity of the constructed model was obtained as 93.6% and 87.3%, respectively with 90.6% classification rate (Supplementary Table 6).

Addition of another orthogonal projection to the above model (Fig. 1C) did not affect the results showing the similar sensitivity and specificity of OPLS-DA model (Supplementary Table 6). ROC plot (Supplementary Fig. 2) and permutation (Supplementary Fig. 3) of obtained OPLS-DA model showed good area under the curve and lesser difference between goodness of fit and predictive ability of model.

The loading plot variables were arranged according to their performance for discrimination among the groups from the validated OPLS-DA model. Figure 1D showed that selenium with highest VIP value is mainly responsible for group separation.

### Metallomic fingerprinting of serum for IHD patients with systolic dysfunction (Ejection fraction (EF) < 45% and EF > 45%)

At the second stage of this study, serum samples of IHD patients were further categorized on the basis of systolic dysfunction. For this purpose, IHD patients were divided into two groups that are, EF < 45% and EF > 45%. Using absolute concentrations of elements, three out of sixteen were found to be significantly different (p < 0.05) (Table 3). Selenium is the only element down regulated in group of patients with EF < 45 and chromium and nickel were found to be up regulated in patients with EF < 45 in comparison to EF > 45.

**Table 3:** List of up-regulated and down-regulated elements in preserved EF > 45% and reduced EF < 45% of systolic dysfunctions.

Elements	p (Corr)	Log FC (SR rEF < 45% vs SR pEF > 45%)	Regulation (SR rEF < 45% vs SR pEF > 45%)
52 Cr	0.049478	0.69644	Up
60 Ni	0.049478	0.471092	Up
78 Se	0.030034	− 0.39566	Down

**Figure 2:**
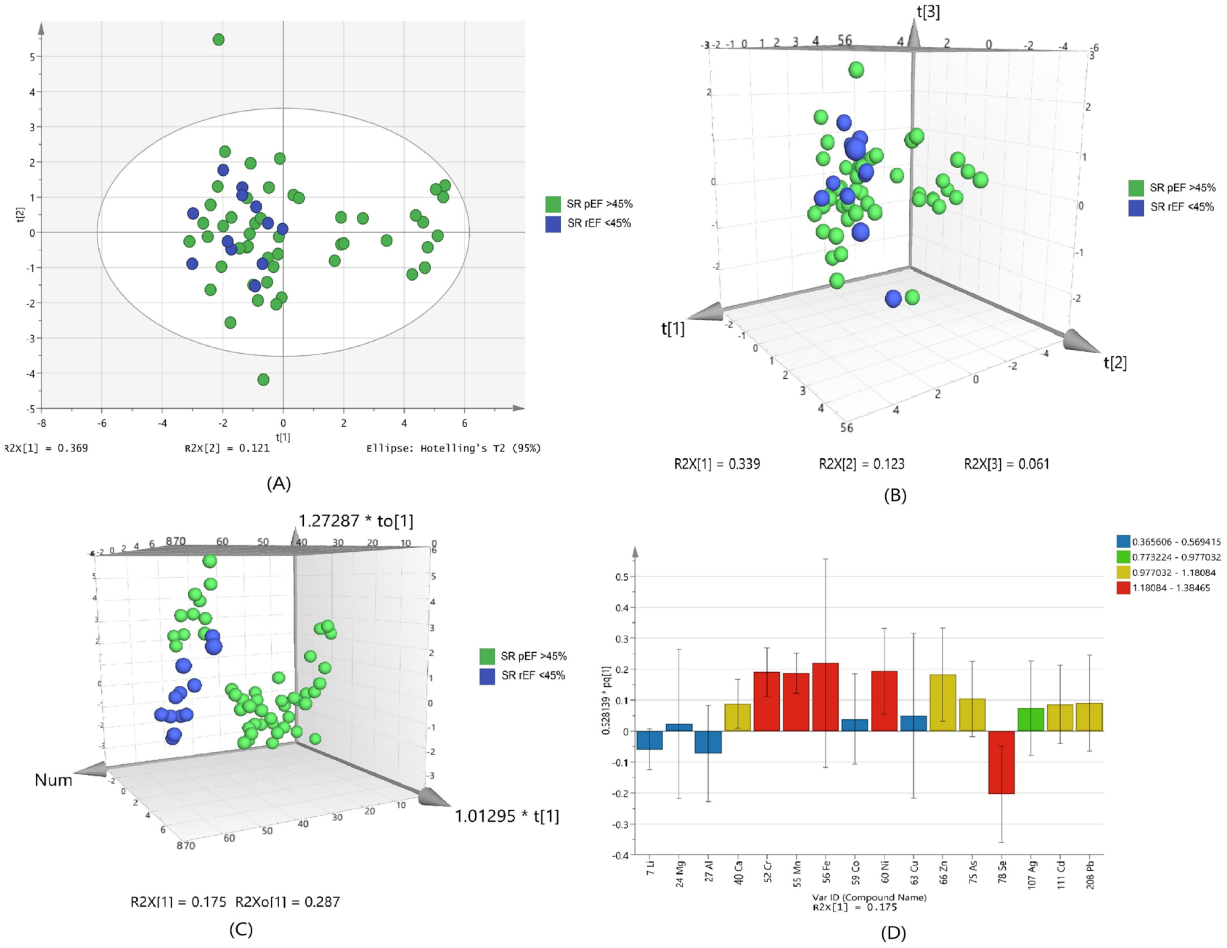
Scores scatter plots (**A**) PCA, (**B**) 3D PLS-DA, (**C**) 3D OPLS-DA and (**D**) OPLS-DA loadings plot of serum with preserved EF > 45% (green) and reduced EF < 45% (blue) of systolic dysfunctions.

Based on the normalized concentration of elements, PCA score plot in Fig. 2A did not show clear separation on the basis of ejection fraction. However, at 95% confidence limit from Hotelling`s T2-test resulted in appearance of fewer outliers as compared to healthy and IHD patient samples. The first component at x-axis gave more variance with the value of R_2_X 0.369 while the second component is 0.121.

For class discrimination, three significant metals were used to perform PLSDA as shown in Fig. 2B. It was found that 3D-PLSDA plot of these metals did not show clear trend of separation in IHD patient with EF > 45% and < 45%. Sensitivity was found to be 98% and specificity was 16.6%, with 82.3% classification rate (Supplementary Table 7). Addition of another orthogonal projection to the above model (Fig. 2C) did not affect the sensitivity and specificity of OPLS-DA model too much (Supplementary Table 7). The reason behind low specificity of the models is clustering of few samples with EF > 45% towards the samples with EF < 45%. The loading plot variables were arranged according to their performance for discrimination among the groups from the validated OPLS-DA model. Figure 2D showed that selenium is mainly responsible for group separation.

### Metallomic fingerprinting of serum for IHD patients with diastolic dysfunction

**Figure 3:**
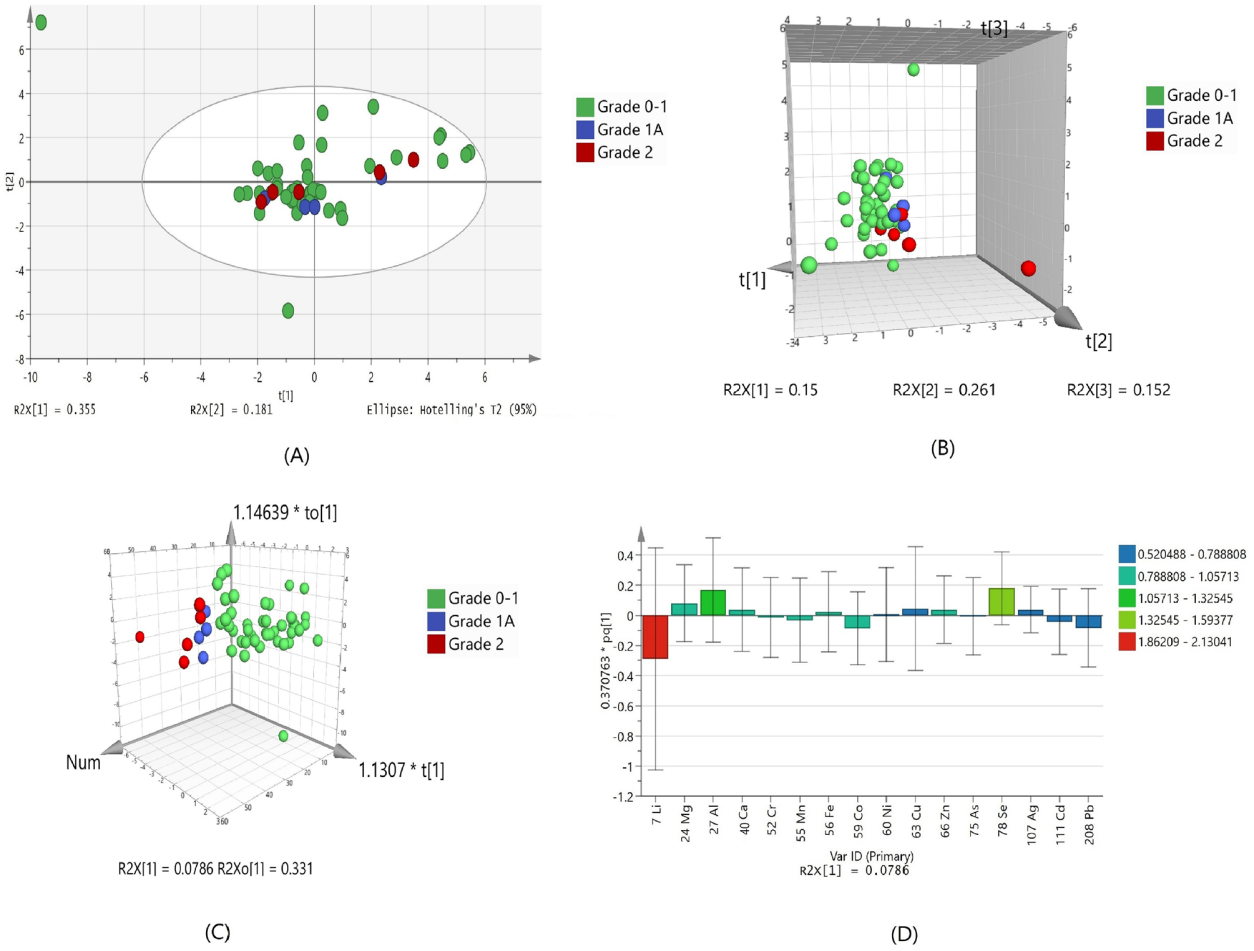
Scores scatter plots (**A**) PCA, (**B**) 3D PLS-DA, (**C**) 3D OPLS-DA and (**D**) OPLS-DA loadings plot of serum with Grade 0–1 (green), Grade 1A (blue) and Grade 2 (red) of diastolic dysfunctions.

The IHD patients in this study had diastolic dysfunction with grade 1, 1A and 2. Statistical significance analysis (ANOVA) resulted in only one significant metal that is selenium; which was down regulated in serum of diastolic dysfunction grade 1A and grade 2 as compared to grade 0–1 (Table 4). PCA showed no separation of groups and only one outlier. This could possibly due to less number of samples in higher grades (Fig. 3A). PLSDA and OPLSDA model (Fig. 3B,C, respectively) were also produce and it shows serum diastolic dysfunction grades were clearly separated and differentiated with each other after orthogonal projection. However, the sensitivity and specificity of the model are 100% and 11%, respectively with 85.2% classification rate (Supplementary Table 7). Due to less number of samples at higher grades of disease, it is unable to generate a specific model as it required. The variable loading plot from the validated OPLS-DA model showed lithium and selenium are mainly responsible for group separation (Fig. 3D).

**Table 4:** list of up-regulated and down regulated elements in serum Grade 0–1, Grade 1A and Grade 2 of dystolic dysfunctions.

Element	p (Corr)	Log FC (SR Grade 1A vs SR Grade 0–1)	Regulation (SR Grade 1A vs SR Grade 0–1)	Log FC (SR Grade 2 vs SR Grade 0–1)	Regulation (SR Grade 2 vs SR Grade 0–1)
78 Se	1.86 × 10^–08^	− 2.1666555	Down	− 0.5374251	Down

Another parameter for measuring diastolic dysfunction by echocardiography includes E/eʹ. The E/eʹ is used as non-invasive surrogate which indicates increased LV filling pressure, for diastolic disfunction^15^. It is measured as the ratio of early mitral inflow and mitral annular early diastolic velocity. The E/e′ of < 8 represents the normal heart filing pressure and increased in the LV filling pressured represented by E/e′ > 15. Another range of E/e′ i.e. 9–15 represents borderline and required additional measurements regarding the confirmation of increased filling pressure.

**Figure 4:**
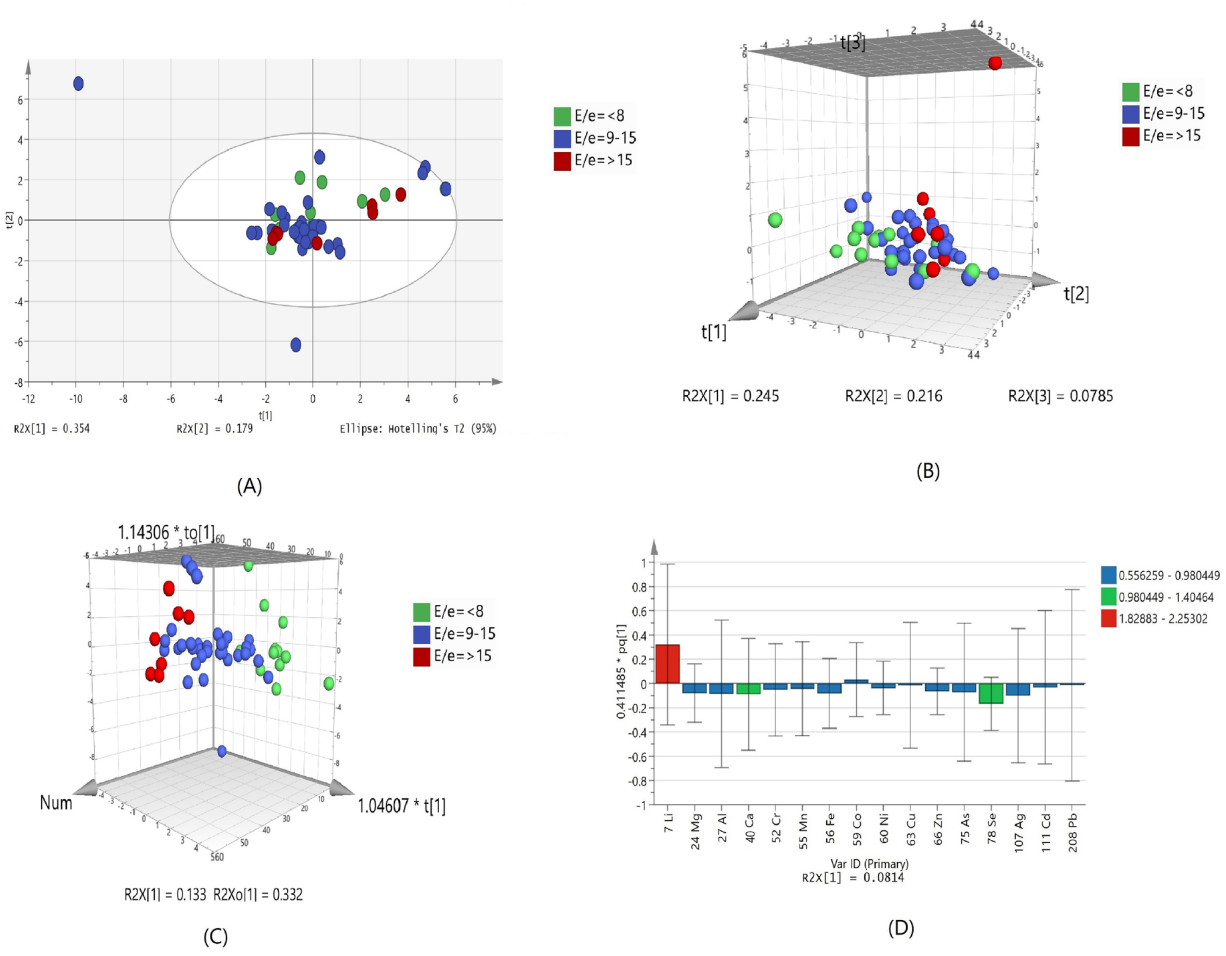
Scores scatter plots (**A**) PCA, (**B**) 3D PLS-DA, (**C**) 3D OPLS-DA and (**D**) OPLS-DA loadings plot of serum with E/eʹ < 8 (green), E/eʹ 9–15 (blue) and E/eʹ > 15 (red) of diastolic dysfunction.

PCA shows no clear separation trend due to the overlapping of samples between groups of IHD patients with different values of E/e′ (Fig. 4A). Significant overlapping is also found in PLSDA model with 33.3% sensitivity and 82.5% specificity (Fig. 4B and Supplementary Table 7). However, OPLSD model (Fig. 4C) showed some trend of discrimination between the three groups. Lithium, selenium and calcium are most important elements, which is related to group differentiation according to the VIP values (Fig. 4D). There is no statistically significant element was found on the basis of E/e′ values.

## Discussion

Our study demonstrated the snap shot of major metals that are differentially regulated in IHD patients in a single canvas using metallomic approach. At first our results described the comparison of serum of IHD patients and healthy subjects. Then the differentially regulated metals in IHD patients with systolic dysfunction grouped in EF > 45 as patients with normal systolic function and EF < 45, as patients with systolic dysfunction were presented. We further analyzed IHD patient based on the different grades of diastolic dysfunction and also looked with respect to their E/e′ ratio, that is the most sensitive indicator for the measurement of higher LV pressures.

Our study has shown that selenium, lithium, aluminum and copper were down regulated in serum of IHD patients compared to healthy subjects, whereas, zinc, silver, arsenic, cadmium and manganese were up regulated in IHD patients. In all of the above mentioned metals, selenium was mainly responsible for group separation.

Previous reports on atherosclerosis observed that the concentration of copper and zinc were significantly lower in patient when compared with healthy, however no correlation was found between the groups and disease severity^16^. Another study suggested the risk factors for acute coronary syndrome might be due to the deficiencies of copper, zinc and selenium^17^.

The trace elements identified in our study as well as in previous work play important roles in cellular and molecular processes in biology^18^. Copper regulates oxidative free radical and its deficiency leads to increased risk of oxidative damage^19^ seen as lipoprotein oxidation^20^, decreased cytochrome C oxidase activity^21^, myofibrillar disarray and mitochondrial impairment^22^. Zinc acts as an antioxidant^23^ and its deficiency has been linked to lipid peroxidation^24^ as well as contractile dysfunction in the heart^25^. Other studies have hinted at the possibility that zinc may increase the risk of IHD^26^. Our results show higher levels of Zn in IHD patients. Serum zinc levels have been shown to correlate with intake of various medications as well as nutritional status, as it has an indirect absorption relationship with copper in IHD patients^26,27^.

Manganese is part of superoxide dismutase and adenylyl cyclase, the two antioxidant enzymes fighting oxidative stress in the body. Mice without the manganese-superoxide dismutase gene die at day 10 of birth due to dilated cardiomyopathy^28^, but its levels were found to be higher in heart failure patients^29^. Cadmium is a non-essential toxic metal found in tobacco, air and food. There is growing evidence that increased levels are associated with high IHD disease risk^30^.

Selenium, an essential trace element, has gathered attention in terms to its importance in the optimal functioning of the cardiovascular system. Selenium mediates its effect through incorporation through various selenoproteins. It has wide ranging effects from regulation of inflammatory response and immune cell activity to control of migration, adherence and phagocytosis of leucocytes^31,32^. Its major contribution, however, is its role in the antioxidant defense mechanism of the body. Selenium is a crucial component of the antioxidant enzyme glutathione peroxidase and thioredoxin reductases. Thioredoxin reductase reproduce the thioredoxin and thus balancing the redox mechanism of the cell, while glutathione peroxidases perform protective mechanism of the cell from DNA damage and/or lipoprotein by detoxifying the intracellular hydrogen peroxide^33^. The platelet aggregation would increase by its deficiency, through the mechanism of free lipid peroxides on prostacyclin synthase and thromboxane A^34^.

It has been postulated that cardiovascular pathologies and low intake of selenium were associated with each other and might be due to the increased in the oxidative stress and its sequelae. Selenium deficiency has been linked historically to Keshan disease, named after a Chinese town where a rapidly progressive cardiomyopathy with degenerative changes and extensive fibrosis in the heart was reported almost 80 years ago^35^. First evidence that selenium contributed to the antioxidant defense mechanism in cardiomyocytes came from Lu et al.^36^ after which a series of animal experiments showed that myocardial injury increased by the deficiency of selenium with lipid peroxidation after myocardial ischemia–reperfusion injuries^37,38^. Experiments have shown convincingly that decreased glutathione peroxidase activity measured in the blood and cardiomyocytes was responsible for this injury^39^. There have been many reports in literature that selenium is associated with risk of IHD^40,41^. Serum selenium was found to be inversely associated with age, smoking, alcohol intake Serum selenium was found to be positively associated with HDL cholesterol, blood hemoglobin, total serum cholesterol and adiposity^42^, and inversely associated with alcohol consumption, smoking and age^43^. Selenium plays vital role in protecting cardiovascular system by forming inactive complexes with metals, such as mercury, cadmium and arsenic which were associated with atherogenesis and resist oxidative damage induced by these metals^44^ and its deficiency has particularly been linked to the degree of myocardial necrosis^45^.

Our study found selenium to be the only element up regulated in patients with EF > 45 as compared to EF < 45. Among patients with IHD, selenium deficiency does correspond to the degree of systolic dysfunction of the heart. Selenium has also come up as an important element in IHD patients as it was also decreases with disease severity in terms of grades of diastolic dysfunction. The patients were grouped according to their diastolic function states into grades, without taking into account their EF status, so our results depict selenium deficiency associated with diastolic dysfunction grade 1A and 2 in comparison to grade 0–1. Our results hint at the common pathophysiological process that play in the development of systolic and diastolic dysfunction of the heart which is associated with selenium deficiency among other trace element perturbations.

## Conclusion

In conclusion, IHD patients with systolic and diastolic dysfunction of the heart showed disturbed trace element profile. Selenium deficiency has been shown to be associated with systolic and diastolic dysfunction of the heart in IHD patients. The phenomena of pathophysiological mechanism involved in the dysfunction of systole and diastole of IHD patients was approached using metallomic fingerprinting of these patients. However, combining these results with metalloproteins analysis in atrial tissue and other biofluids will clear the mechanism in deep. The size of samples in our study is small as one of the limitation and also did not include the high grades of diastolic dysfunction such as Grade III and IV.

## Supplementary information

Supplementary Information
