# Supplementary material for: Understanding of metals dysregulation in patients with systolic and diastolic dysfunction in ischemic heart disease
Source: Sci Rep. 2020 Aug 18;10:13948. doi: 10.1038/s41598-020-70733-4 (PMC7434915; doi:10.1038/s41598-020-70733-4)
Supplement: Supplementary file 1 — Supplementary Information [file 41598_2020_70733_MOESM1_ESM.docx]

**Understanding of metals dysregulation in patients with Systolic and Diastolic dysfunction in Ischemic heart disease**

Noman Khan^1^, Satwat Hashmi^2^, Amna Jabbar Siddiqui^3^, Sabiha Farooq^1^, Shahid Ahmed Sami^4^, Nageeb Basir^5^, Syeda Saira Bokhari^5^, Hasanat Sharif^4^, Sanaullah Junejo^6^, Hesham R. El-Seedi^7,8^, Syed Ghulam Musharraf ^1,3*^

*^1^H.E.J. Research Institute of Chemistry, International Center for Chemical and Biological Sciences, University of Karachi, Karachi-75270, Pakistan*

*^2^Department of Biological and Biomedical Sciences, Agha Khan University, Karachi-74800, Pakistan*

*^3^Dr. Panjwani Center for Molecular Medicine and Drug Research, International Center for Chemical and Biological Sciences, University of Karachi, Karachi-75270, Pakistan*

*^4^Department of Surgery, Aga Khan University, Karachi-74800, Pakistan*

*^5^Department of Medicine, Aga Khan University, Karachi-74800, Pakistan*

*^6^ South City Hospital Karachi-75600, Pakistan*

*^7^Pharmacognosoy Group, Department of Medicinal Chemistry, Uppsala University, Biomedical Centre, Box 574, 75 123 Uppsala, Sweden*

*^8^Alrayan Medical College, Medina 42541, Kingdom of Saudi Arabia*

^*^ Corresponding author. Tel: + 92 21 34824924-5; 34819010; fax: + 92 21 34819018-9.

Email address: [musharraf1977@yahoo.com](mailto:musharraf1977@yahoo.com)

**Supplementary Figure legends**

**Supplementary Figure 1.** PCA plot discrimination on the basis of (A) Age, (B) body weight, (C) BMI, (D) SBP and (E) DBP.

**Supplementary Figure 2.** Receiver operating characteristic (ROC) plot for OPLS-DA model showing sensitivity on y-axis and 1 -specificity on x-axis [SR Healthy (green) and SR IHD (blue)].

**Supplementary Figure 3.** Permutation plots of (A) Healthy serum and (B) IHD serum for the OPLS-DA model showing R2 (green) and Q2 (blue) values.

**
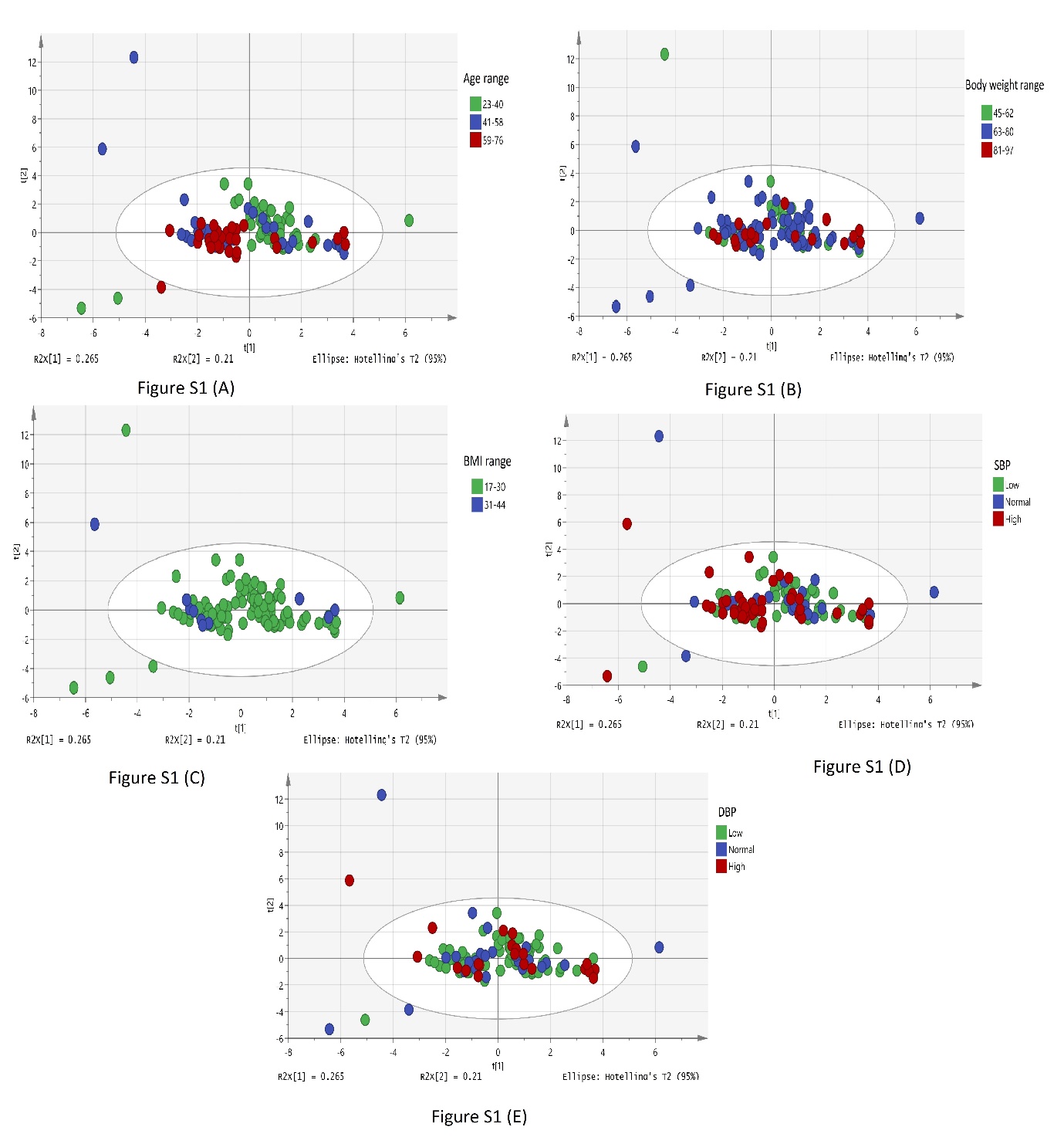
**

**Supplementary Figure 1.** PCA score plot on the basis of (A) Age, (B) body weight, (C) BMI, (D) SBP and (E) DBP.

**
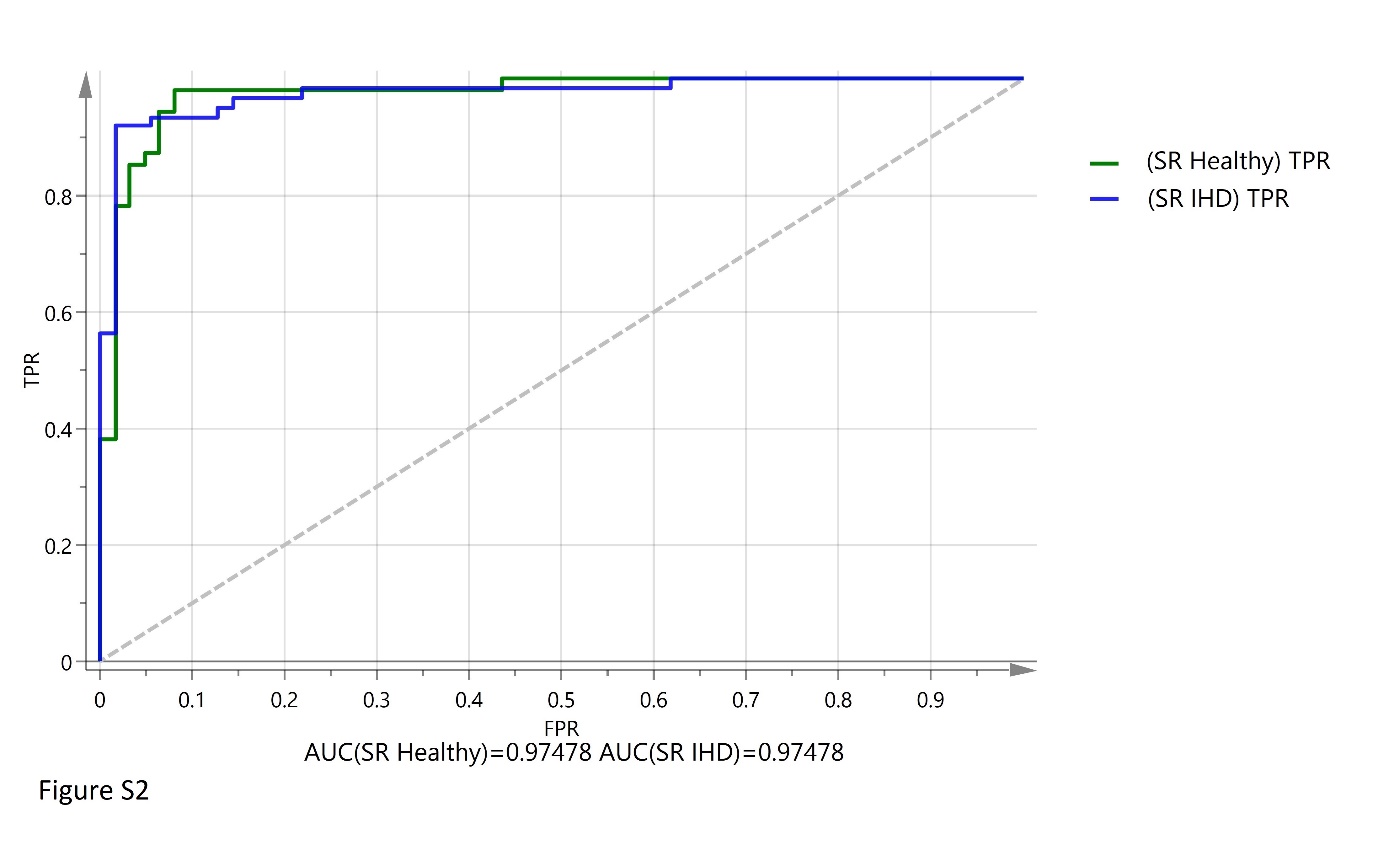
**

**Supplementary Figure 2.** Receiver operating characteristic (ROC) plot for OPLS-DA model showing sensitivity on y-axis and 1 -specificity on x-axis [SR Healthy (green) and SR IHD (blue)].

**
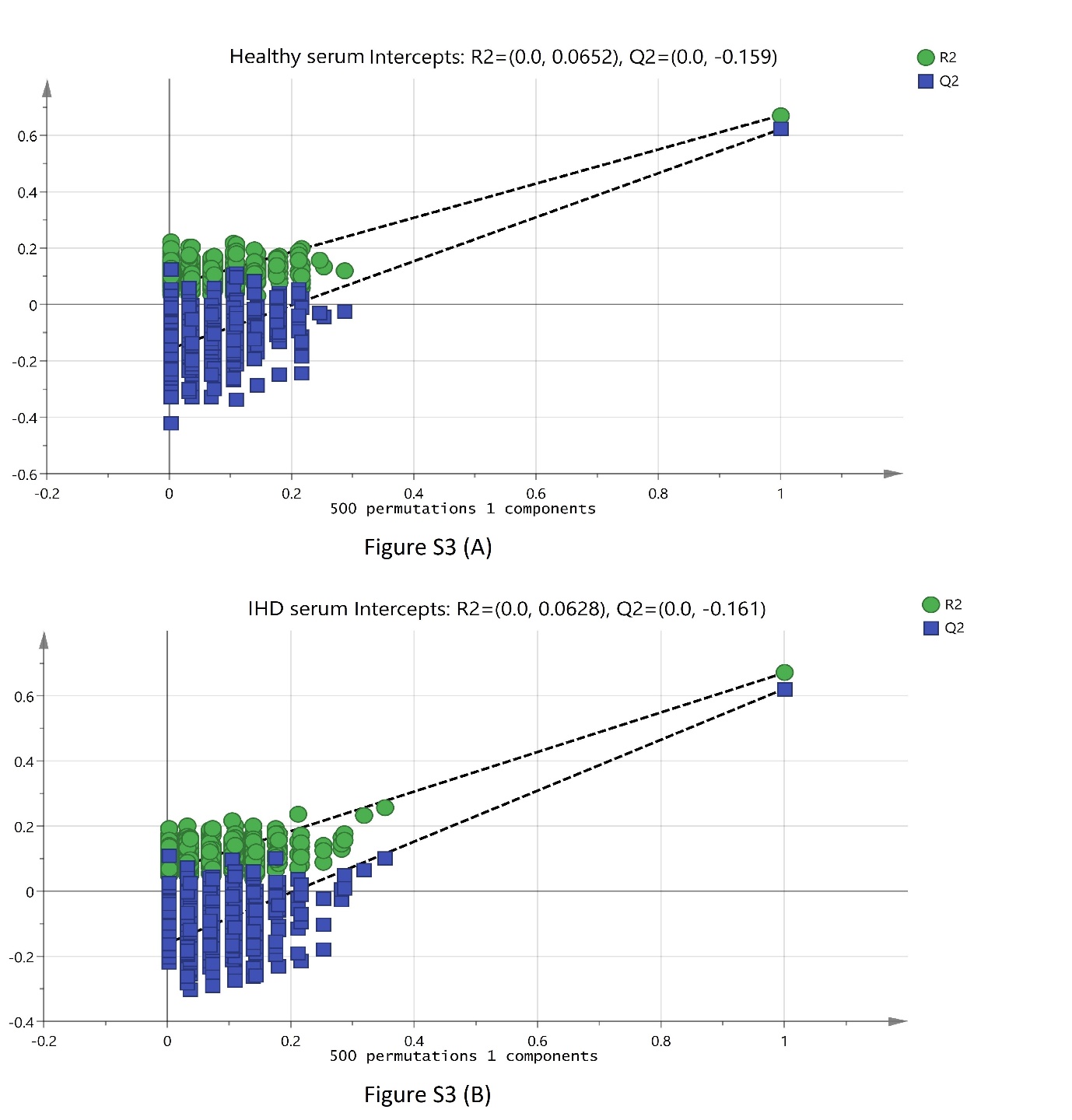
**

**Supplementary Figure 3.** Permutation plots of (A) Healthy serum and (B) IHD serum for the OPLS-DA model showing R2 (green) and Q2 (blue) values.

**Supplementary Table legends:**

**Supplementary Table 1:** Clinical differences between IHD patients with EF <45% and > 45%, and in those with diastolic dysfunction

**Supplementary Table 2.** ICP-MS operating conditions.

**Supplementary Table 3.** Summary of ICP-MS calibration.

**Supplementary Table 4.** Concentration of the elements observed in certified standard reference material (n = 3).

**Supplementary Table 5.** Validity of the proposed method in two different concentration levels.

**Supplementary Table 6.** Average prediction results obtained by a default method of 7-fold internal cross validation of the software of PLS-DA and OPLS-DA models based on elemental analysis of serum healthy and IHD patients.

**Supplementary Table 7.** Average prediction results obtained by a default method of 7-fold internal cross validation of the software of PLS-DA and OPLS-DA models based on elemental analysis of IHD patients categorized on the basis of echocardiographic parameters.

**Supplementary Table 1:** Clinical differences between IHD patients with EF <45% and > 45%, and in those with diastolic dysfunction

| **Systolic dysfunction (Ejection fraction <45% and >45 %)** | | | | |
| --- | --- | --- | --- | --- |
|  | | EF<45 | EF>45 | p-value |
| Creatinine (mg/dl) | | 1.11±0.34 | 0.96±0.23 | 0.211 |
| BUN (mg/dl) | | 20.45±12.11 | 16.81±6.27 | 0.404 |
| Random blood glucose (mg/dl) | | 212.25±56.07 | 184.37±66.57 | 0.676 |
| Fasting blood glucose (mg/dl) | | 191±88.19 | 154±41.70 | 0.358 |
| **Diastolic dysfunction (grade 0-1, 1A and 2)** | | | | |
|  | Grade 0-1 | Grade 1A | Grade 2 |  |
| Creatinine (mg/dl) | 1.00±0.25 | 0.67±0.23 | 1.05±0.33 | 0.089 |
| BUN (mg/dl) | 16.10±5.67 | 18±14.14 | 22.58±4.30 | 0.132 |
| Random blood glucose (mg/dl) | 185.82±66.57 | 144±5.66 | 220±56.57 | 0.449 |
| Fasting blood glucose (mg/dl) | 168.04±58.95 | 134.5±21.92 | 135±21.21 | 0.941 |

**Supplementary Table 2.** ICP-MS operating conditions.

| **Plasma Parameter** | | **Ion Lenses** | |
| --- | --- | --- | --- |
| RF power | 1600 W | Extract 1 | 0.0 V |
| RF matching | 1.80 V | Extract 2 | -195.0 V |
| Sample depth | 10.0 mm | Omega bias | - 80.0 V |
| Carrier gas | 0.36 L min^-1^ | Omega lens | 8.6 V |
| Nebulizer pump | 0.10 rps | Cell entrance | -40 V |
| Spray chamber temperature | 2°C | Cell exit | -60 V |
| Dilution gas | 0.63 L min^-1^ | Deflect | 1.4 V |
| Nebulizer | MicroMist | Plate bias | -60 V |
| Plasma Ar gas flow rate | 15 L min^-1^ | **Reaction Cell** | |
| Sample uptake rate | 0.30 rounds per second | He flow | 5 mL min^-1^ |
| Sample uptake volume | 1.0 mL | **Q-Pole Parameters** | |
| Acquisition time | 5 min | OctP bias | -18.0 V |
| Integration time | 1 s^-1^ | OctP RF | 180 V |
|  |  | Energy discrimination | 5.0 V |

**Supplementary Table 3.** Summary of ICP-MS calibration.

| **Metal** | **Mass** | **Regression equation** | **R^2^** | **LOD (µg L^-1^)** | **LOQ (µg L^-1^)** |
| --- | --- | --- | --- | --- | --- |
| Li | 7 | y=883.8823x+2973.6133 | 0.995 | 0.359 | 1.089 |
| Mg | 24 | y=3239.6092x+25484.1400 | 0.998 | 0.060 | 0.182 |
| Al | 27 | y=5.9529x+51.6667 | 1.000 | 5.172 | 15.673 |
| Ca | 40 | y=3.7195x+354.6767 | 1.000 | 5.490 | 16.637 |
| Cr | 52 | y=557.0202x+245.6667 | 1.000 | 0.022 | 0.068 |
| Mn | 55 | y=181.5825x+40.0000 | 1.000 | 0.099 | 0.300 |
| Fe | 56 | y=380.7498x+1266.7200 | 1.000 | 0.168 | 0.510 |
| Co | 59 | y=1173.7717x+28.3333 | 0.999 | 0.026 | 0.078 |
| Ni | 60 | y=313.4538x+879.0300 | 1.000 | 0.442 | 1.339 |
| Cu | 63 | y=893.1232x+901.0300 | 1.000 | 0.094 | 0.285 |
| Zn | 66 | y=92.7534x+1129.0400 | 1.000 | 0.700 | 2.120 |
| As | 75 | y=68.3991x+5.5000 | 1.000 | 0.088 | 0.266 |
| Se | 78 | y=2.1143x+2.3333 | 1.000 | 2.167 | 7.071 |
| Ag | 107 | y=1558.2113x+27.000 | 0.993 | 0.002 | 0.006 |
| Cd | 111 | y=116.1793x+16667 | 1.000 | 0.039 | 0.120 |
| Pb | 208 | y=1832.1951x+1230.0533 | 0.988 | 0.068 | 0.207 |

**Supplementary Table 4.** Concentration of the elements observed in certified standard reference material (n = 3).

| **S. No.** | **Analyte** | **Reference values**  **(µg L^-1^)** | **Found values**  **(µg L^-1^)** | **% Recovery** |
| --- | --- | --- | --- | --- |
| 1 | Li | 5741 ± 321 | 5740.458 ± 17.636 | 99.990 |
| 2 | Mg | 20600 ± 1000 | 23438.486 ± 1813.249 | 113.779 |
| 3 | Al | 33.6 ± 1.9 | 35.286 ± 0.500 | 105.017 |
| 4 | Ca | 94200 ± 4400 | 77130.440 ± 4506.766 | 81.879 |
| 5 | Cr | 1.5 ± 0.2 | 1.439 ± 0.313 | 95.929 |
| 6 | Mn | 15.0 ± 0.9 | 15.141 ± 0.682 | 100.938 |
| 7 | Fe | 1430 ± 8.0 | 1441.360 ± 10.523 | 100.794 |
| 8 | Co | 1.2 ± 0.2 | 1.269 ± 0.166 | 105.782 |
| 9 | Ni | 5.8 ± 0.7 | 6.127 ± 0.165 | 105.638 |
| 10 | Cu | 1691 ± 84 | 1636.041 ± 89.144 | 96.750 |
| 11 | Zn | 1742 ± 82 | 1738.928 ± 85.630 | 99.824 |
| 12 | As | 0.47 | 0.456 ± 0.005 | 97.051 |
| 13 | Se | 107 ± 7 | 107.237 ± 5.311 | 100.221 |
| 14 | Ag | 0.16 | 0.155 ± 0.018 | 96.731 |
| 15 | Cd | 0.126 | 0.129 ± 0.067 | 102.535 |
| 16 | Pb | 1.02 | 1.056 ± 0.061 | 103.514 |

**Supplementary Table 5.** Validity of the proposed method in two different concentration levels.

| **S. No.** | **Elements** | **Serum samples** | | | |
| --- | --- | --- | --- | --- | --- |
|  |  | **Concentrations C_E_ (µg L^-1^)** | **Calculated concentrations**  **C_M_ (µg L^-1^)** | **Precision (% RSD)** | **% Recovery** |
| 1 | 7 Li | 50 | 52.820 | 7.580 | 104.047 |
|  |  | 100 | 102.932 | 2.648 | 102.135 |
| 2 | 24 Mg | 50 | 382.167 | 1.296 | 95.560 |
|  |  | 100 | 434.187 | 1.066 | 99.800 |
| 3 | 27 Al | 50 | 184.055 | 0.052 | 104.069 |
|  |  | 100 | 284.814 | 2.828 | 152.793 |
| 4 | 40 Ca | 50 | 459.324 | 0.376 | 41.138 |
|  |  | 100 | 461.230 | 2.635 | 22.475 |
| 5 | 52 Cr | 50 | 55.228 | 1.107 | 97.634 |
|  |  | 100 | 105.094 | 0.665 | 97.582 |
| 6 | 55 Mn | 50 | 51.349 | 2.490 | 97.954 |
|  |  | 100 | 103.000 | 1.152 | 100.975 |
| 7 | 56 Fe | 50 | 134.266 | 1.634 | 100.879 |
|  |  | 100 | 194.137 | 1.357 | 97.968 |
| 8 | 59 Co | 50 | 57.539 | 1.686 | 112.847 |
|  |  | 100 | 114.814 | 1.080 | 113.773 |
| 9 | 60 Ni | 50 | 54.366 | 1.879 | 95.584 |
|  |  | 100 | 114.206 | 1.152 | 108.347 |
| 10 | 63 Cu | 50 | 74.028 | 2.009 | 106.858 |
|  |  | 100 | 132.032 | 0.722 | 112.710 |
| 11 | 66 Zn | 50 | 94.056 | 1.419 | 34.560 |
|  |  | 100 | 125.367 | 1.070 | 48.591 |
| 12 | 75 As | 50 | 50.484 | 2.604 | 100.834 |
|  |  | 100 | 100.184 | 0.912 | 100.017 |
| 13 | 78 Se | 50 | 45.149 | 7.169 | 88.355 |
|  |  | 100 | 100.700 | 6.818 | 99.313 |
| 14 | 107 Ag | 50 | 57.896 | 1.753 | 115.315 |
|  |  | 100 | 105.555 | 4.707 | 105.224 |
| 15 | 111 Cd | 50 | 50.073 | 1.941 | 100.105 |
|  |  | 100 | 99.821 | 0.435 | 99.766 |
| 16 | 208 Pb | 50 | 47.944 | 1.666 | 90.953 |
|  |  | 100 | 97.692 | 1.852 | 95.154 |

**Supplementary Table 6.** Average prediction results obtained by a default method of 7-fold internal cross validation of the software of PLS-DA and OPLS-DA models based on elemental analysis of serum healthy and IHD patients.

|  | **R2** | **Q2** | **Sensitivity** | **Specificity** | **Classification rate** |
| --- | --- | --- | --- | --- | --- |
| PLS-DA model | | | | | |
|  | 0.363 | 0.599 | 87.27% | 93.55% | 90.6% |
| OPLS-DA model | | | | | |
|  | 0.363 | 0.621 | 87.27% | 93.55% | 90.6% |

**Supplementary Table 7.** Average prediction results obtained by a default method of 7-fold internal cross validation of the software of PLS-DA and OPLS-DA models based on elemental analysis of IHD patients categorized on the basis of echocardiographic parameters.

| **Model** | **R2** | **Q2** | **Sensitivity** | **Specificity** | **Classification rate** |
| --- | --- | --- | --- | --- | --- |
| **pEF >45% and rEF <45%** | | | | | |
| PLS-DA | 0.523 | 0.312 | 98% | 16.67% | 82.26% |
| OPLS-DA | 0.462 | 0.286 | 96% | 25% | 82.26% |
| **Grades of IHD patients** | | | | | |
| PLS-DA | 0.563 | -0.12 | 100% | 20% | 85.19% |
| OPLS-DA | 0.409 | -0.0494 | 100% | 20% | 85.19% |
| **E/e´ of IHD patients** | | | | | |
| PLS-DA | 0.54 | -0.0974 | 33.33% | 82.5% | 71.15% |
| OPLS-DA | 0.465 | -0.0575 | 41.67% | 77.5% | 77.5% |
